# Some Practical Observations in Pelvic Operations

**Published:** 1897-07

**Authors:** J. H. Sampson

**Affiliations:** Houston, Texas


For the Texas Medical Journal.

### SOME PRACTICAL OBSERVATIONS IN PELVIC OPERATIONS.

BY J. H. SAMPSON, M. D., HOUSTON, TEXAS.

[Read before the South Texas Medical Association, at Galveston, May 14th, 1897.]

THAT the pendulum in some forms of gynecological work has been receding for the past four or five years from the extreme limits that had been reached by very radical procedure, is quite evident. Some of these extreme radical measures have had to commend them only selfish motives, wherein the chief object was to be credited with having done a laparotomy, or the compiling of the statistics of many laparotomies with the minimum of fatalities. We can not too highly esteem the stand taken by such men as Wm. M. Polk, Howard A. Kelly, Prior, and surgeons of their stamp, in combatting this craze that resulted in the unnecessary mutilation of so many women. Heretofore certain pathological conditions of the ovaries and tubes wherein complete extirpation was at once resorted to, we now find, by a conservative and more rational procedure, that these essential organs can be saved and health restored. In achieving this marked advancement in pelvic surgery, vaginal section or colpotomy has played a most important part. We note that in 1813, Langenbach selected the vaginal route for hysterectomies, but it met with disfavor on account of the high mortality, hence vaginal section is not an operation of such recent origin. In 1878, we find Czerney, with the proper antiseptic precaution, reestablishing this route of surgical procedure, and thus placing it upon a sound foundation. This operation, within the past few years, has taken such hold upon some of our leading gynecologists, that it has almost entirely supplanted the abdominal route in pelvic operations. We will consider some of the advantages that colpotomy presents over the abdominal route:

First. As to surgical shock during and following this method of operating, our experience has been most gratifying, as this danger has been scarcely manifested. We offer these suggestions as an explanation, viz: the extent of wound and amount of trauma to the bowels that specially manifests its effect upon the sympathetic system in the abdominal operation.

The second advantage is that of drainage, recognizing that the incision is located in the most dependent portion of the abdominal cavity.

Third. This locality, by some wise provision of nature, seems either less vulnerable or of a greater power of resistance to septic invasion than other portions of the peritoneal cavity, for herein there is a tendency well marked to localize all inflammatory processes.

Fourth. In regard to ligature, it is very rarely that a ligature is called for (with the exception of precautionary ligature used by us, which is easily removed along with the clamps), thus leaving the abdominal cavity free from any foreign body.

Fifth. Freedom from interference with the bowels.

Sixth. As to suturing the incision, this is not necessary.

Seventh. Freedom from interference with field of operations after the first dressing of the case, thus much is added to the comfort of the patient during convalescence.

Eighth. The rapidity of convalescence due to the above named advantages is certainly a strong point in favor of this operation.

Ninth. The freedom from unsightly scar.

Tenth. Freedom from hernia. So prone is hernia to follow laparotomies, that we find Howard Kelley advocates closing the abdominal incision with silver wire mattresses as a necessary precaution. Where the operation is done within the limits indicated, we know of no case of hernia following the vaginal method.

THE INDICATIONS FOR THE VAGINAL SECTIONS IN PELVIC OPERATIONS.

It should be the aim and pride of the surgeon to preserve everything consistent with thorough surgery, and not to sacrifice important organs simply because it can be done without increasing the mortality. A slow convalescence, or even a second operation, is certainly preferable to unnecessary mutilation, and it is through this route that tubes and ovaries may be saved by being locally treated and properly drained, where in the abdominal operation extirpation would be unavoidable. It is true that many gynecologists of high standing advocate the removal of the uterus in all cases where the removal of its appendages has been found necessary.

This, to me, seems anything but rational theory, even though the womb has been deprived of its most important functionating powers in losing its appendages. Its presence, however must certainly have a good mental influence, if only from a sexual standpoint.

Second. For diagnostic purposes.—By colpotomy we so satisfactorily come in direct touch and view of the pelvic organs that have held our method of procedure in doubt and suspense, that the minimum of danger in this exploratory incision makes the venture fully justifiable.

Third. For liberating posterior uterine adhesions in doing the Alexander operation in retroversion, also the liberation of adhesions of the tubes and ovaries.

Fourth. For meeting and aborting pelvic inflammation affecting the tubes and ovaries. As to severe inflammations elsewhere, we find free incision and drainage has its beneficial influences in colpotomy. By a timely interference under this head, we can abort serious suppurative processes with all their ugly consequences, and thereby save a long siege of suffering, and perhaps continued invalidism.

As Prof. Montgomery, of Jefferson, states, you may accomplish by this intervention in a week, that which tentative treatment and application will not do in years, and perhaps then leave your patient a confirmed invalid.

Fifth. For opening and draining, to the best advantage, pelvic abscesses, including pyosalpynx, peritubal and ovarian abscesses, also hydrosalpynx, hæmmatosalpynx, and ovarian cysts.

Sixth. For removal of small subserous fibroids, also papalomotous and other growth of the ovaries.

Seventh. Extra uterine pregnancy in its early stage.

Eighth. For hysterectomy, where we have malignant conditions confined to the uterus, or prolapse of such severe degree as to justify the operation.

Ninth. For removal of ovaries and tubes.

The contra indications for the vaginal route.

First. Large fibromata, miomata, and malignant tumors which involve adjacent tissues.

Secondly. Where there exists a large extent of inflammatory adhesions involving adjacent abdominal viscera. The degree of these contra-indications, however, depends, to a great extent, upon the individual operator. As we well know, some of our best gynecologists resort to vaginal route for removing large fibroids and miomatous growths by morcelation. Others, however, do not take so kindly to this method. I, myself, have seen fatal results from morcelation in a large miomata in the hands of one of our representative gynecologists of America on account of not being able to control the hemorrhage. The obscurity of the field of operation appeared to me like too much grouping in the dark, and I must say I am not favorably impressed with this mode of procedure.

In regard to methods of operating. This is governed entirely by the individuality of the case; hence we will not go into any detail here, as sufficient literature has already been placed before the profession.

A short report of some of the more interesting points that have come under our personal observation may be here of interest:

Mrs. D., nullapara. An epithelioma of the cervix.

Thinking here that the malignancy only involved the cervix, its amputation was all we thought necessary. After making the operation, we discovered that the condition extended into the body of the womb; hence, complete hysterectomy had to be done. By the vaginal route this was easily and quickly accomplished. However, what was an interesting and valuable lesson to us in this case was the treatment of the appendages. We used ligatures as hæmostatics. These ligatures were the only annoyance we experienced in the progress of the case, making it necessary for their complete removal about the sixth day.

Mrs. M., multipara, aged about 45; general nutrition, bad; long existing prolapse for years; the cervix protruding beyond the vulva, and with indications of malignancy. There was a history of an old pelvic peritonitis, also of ugly bladder symptoms. We entered into this operation impressed with the simplicity of the case for a vaginal hysterectomy. Upon opening into the peritoneal cavity, posteriorly, we were confronted by a very pronounced elongation of the supra-vaginal portion of the cervix, complicated with severe adhesions. The anterior cul-de-sac was completely obliterated by strong adhesion between the bladder and uterus, thus necessitating a very careful dissection of at least three inches along the anterior surface of the uterus. With all these complications, forty minutes was consumed in doing this operation. This case clearly demonstrates to us the advantages gained by the vaginal method. In this case ligatures were again used, but to no advantage, and with this we entirely discarded the use of ligatures in these operations, excepting a precautionary ligature which is passed through the broad ligament and left with a long loop for traction, in case the clamps should slip. Another interesting point in the after-treatment also occurred in this case. It is our custom, after removal of clamps and dressing, to begin on about the third day irrigating or washing the vulva and lower vaginal surfaces with a saturated boric acid solution. In this case the nurse permitted the patient to use the irrigator herself, which resulted in her allowing quite a quantity of fluid to be thrown directly into the abdominal cavity by forcing the nozzle of the syringe through the incision. The shock that immediately followed was quite pronounced—the patient's condition becoming critical. The advantage of this method of drainage was here also well demonstrated—a piece of iodoform gauze was at once carried into the abdominal cavity through the incision, and was undoubtedly the essential element in bringing this patient around; notwithstanding this accident and the extensive operative procedure, the patient returned to her home in the third week. The rapid closing and contraction of these wounds will allow careful irrigation; but we should not use enough pressure to force a strong flow against the upper wall.

Mrs. E. B., nullapara.—When first seen, patient presented marked symptoms of advanced pyæmia. A very large tumor distended the iliac region. Diagnosis, pyosalpynx. Preparations were at once made for operation. Short time previous to operation patient showed evidence of escape of contents of tumor into the peritoneal cavity. Laparotomy was at once proceeded with. Upon opening the abdominal cavity a large quantity of very offensive pus was found; the peritoneal cavity was thoroughly washed out with sterilized water, the abscess cavity being well cleansed and wiped out with iodoform gauze; the patient at this time seemed almost moribund. Thinking the only hope for recovery depended upon thorough drainage, posterior colpotomy was also resorted to and iodoform gauze carried from the seat of the abscess out through the vaginal opening. To the surprise of all who witnessed this case the patient recovered. This result should be attributed to the thorough system of drainage.

In order that we may demonstrate the advisability of colpotomy in extensive inflammatory conditions in the pelvis, I will report two cases:

Miss M. P., virgin.—After suffering for many years from the result of inflammatory conditions surrounding the womb, tubes and ovaries, following severe cold from imprudence during menstruation. After tentative treatment for some time, we resorted to this method for relief, with the most gratifying results. Upon operation we found considerable adhesion surrounding both tubes and ovaries. The uterus was also retroflex and bound down by adhesion.

The adhesions were broken up and the organs liberated. The field of operation was drained with iodoform gauze, Alexander's operation also being performed. The patient made a rapid and complete recovery, and has for the past two years been in perfect health.

The second case of this kind was in Mrs. S., multipara. This case also interestingly demonstrated the importance of exploratory incision in diagnosis. Patient suffered severely in the right ovarian region. Careful digital examination with bimanual palpation warranted a diagnosis of prolapsed and congested right ovarian tube. Upon opening into the peritoneal cavity through a posterior vaginal incision, the tumor which was supposed to be a prolapsed ovary was found to be a mass of fat, which was no doubt appendices epiploicar connected with upper portion of rectum. The ovary and tube, however, were found very much congested, and with many adhesions. The organs were liberated by breaking up of the adhesions; also several papalomatous growths were removed from the ovary and perinorrhphy being done at same sitting. This patient made a rapid recovery, and since then has never complained.

In order that we may demonstrate the important advantages gained over laparotomy in the rapidity of convalescence, I offer this case:

Mrs. G., nullapara, aged 21, very sensitive prolapsed and congested ovarian tube, which had formerly been reposited by similar operation. The removal of these organs consumed about twenty-five minutes. This patient was out of bed and sitting up on the fifth day, and left for her home before the end of the second week, the wound being entirely healed.

Mrs. N.—A chronic pelvic abscess existing for some years. When examined by us the tumor had taken on almost a cast of the pelvis, and with the long standing indurated inflammatory condition of the pelvic tissues gave, upon examination, the idea of malignancy. However, we were not positive in our diagnosis, and the laparotomy was considered advisable in order to clear up diagnosis; and was performed to our sorrow. Upon entering the abdominal cavity the true status of affairs presented itself. We found ourselves dealing with a very large pelvic abscess which was so rotten that it seemed to rupture spontaneously; many coils of intestines were adhered and matted down, and the whole cavity seemed to be transformed into an abscess. We washed and dried it out as clean as possible under the circumstances, but the patient we lost. I firmly believe that an exploratory vaginal incision or aspiration simply for the benefit of dignosis, followed by thorough opening, irrigating and draining through the vagina would have saved this patient's life.

Within the week we were compelled to operate upon a ventral hernia that resulted from an ovariotomy through an abdominal section, which was closely followed by several pregnancies. We found very extensive adhesions existing around the site of hernia, which caused much distress. From our experience in private practice, which includes six hysterectomies by the vaginal route, and eight by the abdominal route, twelve oophorectomies by the vaginal and eighteen by the abdominal route, also those cases in which we found colpotomy necessary in order to liberate the uterus, tubes and ovaries previous to performing Alexander's operation. We have also upon several occasions performed perineorrhaphy along with colpotomy at the same sitting, with perfect results.

We can but conclude that many ugly conditions and complications may be obviated by this method of procedure. The limited time allotted to this paper will not admit of my going into detail as to the many interesting points to be observed in pelvic operations, but I trust sufficient has been shown to sustain the advantages that the vaginal route for most pelvic operations so justly merits.

